# Ethnic discrimination in Scandinavia: evidence from a field experiment in women’s amateur soccer

**DOI:** 10.1057/s41599-023-01734-7

**Published:** 2023-05-11

**Authors:** Rasmus K. Storm, Cornel Nesseler, Marthe Holum, Andreas Nygaard, Tor Georg Jakobsen

**Affiliations:** 1 Danish Institute for Sports Studies, Aarhus, Denmark; 2 Department of Social Sciences, University of Stavanger, Stavanger, Norway; 3 Department of Computer Science, Norwegian University of Science and Technology, Trondheim, Norway; 4 NTNU Business School, Norwegian University of Science and Technology, Trondheim, Norway; 5 Present Address: NTNU Business School, Norwegian University of Science and Technology, Trondheim, Norway

**Keywords:** Social policy, Politics and international relations

## Abstract

In this paper, we examine ethnic discrimination using sport as a laboratory. Applying a field experiment in the three Scandinavian countries—Sweden, Norway, and Denmark—we test whether foreign female minority groups experience greater rejection rates when seeking inclusion in amateur soccer clubs. Soccer coaches were contacted by e-mail using native and foreign-sounding names from selected groups, requesting to participate in trial practice. Previous findings show persistent discrimination of foreign minority groups in the labour market, and recent work suggests that discrimination also occurs in the context of soccer. Our findings from Scandinavia show that Sweden is the only country that shows statistically significant signs of discriminatory patterns, and the probability of experiencing discrimination increases with cultural distance. However, cultural distance appears to have no influence in Norway and Denmark. We further investigate whether male or female coaches demonstrate different discriminatory behaviour when being contacted, but our analysis shows almost no gender differences. Findings suggest that how men and women differ in their discriminatory behaviour is context specific. The differences identified across nations and previous studies are discussed to better understand the mechanisms of discrimination.

## Introduction

Social integration of immigrants and minority groups is crucial for flourishing societies (Portes, 1997). It is also important to the individual because an absence of inclusion can lead to difficulties in their everyday lives, such as lacking a sense of belonging, family crises, depression, and even suicide attempts (Lewin, 2001).

This study investigates ethnic discrimination in women’s amateur soccer in Sweden, Denmark and Norway. When it comes to minority women in sports, there has historically been a lack of coverage in media and research (Bruening, 2005; Gomez-Gonzalez et al., 2021; Lovell, 1991). However, in recent decades, a larger body of research has examined their participation (e.g., Elling and Knoppers, 2005; Engh et al., 2017; Ratna, 2011). Focusing on sport in relation to minorities and women is essential because organised sports activities in clubs can potentially be an essential sphere for inter-ethnic socialisation, forging identities and bringing people together (Spaaij, 2011). Leisure contact, in general, between immigrants and people in the local population is argued to be one of the most critical factors in reducing prejudicial attitudes (McLaren, 2003; Pettigrew, 1998). While there is scant research in this particular context, a recent field experiment found evidence of ethnic discrimination in men’s soccer clubs in Denmark, Norway, and Sweden, among other countries (Gomez-Gonzalez et al., 2020).

However, these findings do not automatically predict that discrimination will occur in the context of women’s soccer. The social categories of gender and ethnicity intersect (Rosette et al., 2018), and should therefore be considered in relation to each other. This can be enabled by studying ethnic discrimination against women in sport—on which there is still little research.

Soccer is chosen as our experimental setting because it is a popular sport among both women and men. Although it was traditionally seen as a men’s sport, women’s participation in soccer has been on the rise in Sweden (Hjelm and Olofsson, 2003; Kjær and Agergaard, 2013), Norway (Skogvang, 2019; Strandbu et al., 2019), and Denmark (Bennike et al., 2020; Storm and Holum, 2021). As well as having a steady growth in participation rates, women’s soccer has also experienced increased professionalisation at the management level. Further, an increased proportion of elite foreign players joining Scandinavian clubs has been observed (Agergaard et al., 2013), indicating that women’s soccer is a potential integration area for minority groups.

These three Scandinavian countries are historically uniform (Necef, 2001) and tolerant, adhering firmly to principles of gender equality, human rights, and international solidarity (Gammelsaeter, 2009; Sawyer and Habel, 2014). Based on this, it could be expected that soccer clubs in Scandinavia would be predominantly accepting of foreigners. However, several studies show that ethnic discrimination is a persistent and extensive challenge in the Scandinavian countries, particularly in the labour market (Åslund and Skans, 2012; Carlsson and Rooth, 2007; Dahl and Krog, 2018; Quillian et al., 2019; Rydgren, 2004). This suggests that there are likely to be instances of discrimination also in sports settings. Although the three countries have many similarities, they are also different in some respects, and country differences in discrimination can be significant (Quillian, 1995). We, therefore, explore the differences between countries instead of treating them as a single unit.

To investigate potential ethnic discrimination, we use rejection or acceptance to requests for participation in trial practice for amateur soccer clubs (Dietl et al., 2021; Nesseler et al., 2019). In the experiment, we contacted clubs from e-mail accounts with either a native- or a foreign-sounding name to test the response across the different groups. Receiving a negative response can be synonymous with not only individual but also structural discrimination.

## Background

Immigration increased in Sweden, Denmark, and Norway from the 1960s, and today around 20 percent of the total population in Sweden, 14 percent in Norway and 12 percent in Denmark are immigrants or descendants of immigrants.^1^ Even though the literature indicates that the integration of minority groups takes time (Gordon, 1961), it is likely that the majority groups in these countries would be accustomed to seeing minorities as a natural part of their respective communities. A more significant proportion of ethnic minority groups increases the possibility of contact between the different groups and, in turn, the likelihood for friendships to arise. This kind of positive interaction is expected to reduce prejudice towards minority groups (Allport, 1954; Blommaert et al., 2014; McLaren, 2003). On the other hand, if segregation occurs, causing reduced or negative interaction, the effect can be the opposite (Pettigrew, 2008).

Sweden has received considerable attention for its challenges associated with immigrants being involved in criminal activity and social disruption (Adamson, 2020; Beckley et al., 2017; Hallsten et al., 2013; Martens, 1997). Viewing the situation in light of research on discrimination and stereotyping, it is likely that discrimination creates a vicious circle starting with overestimating in-group and out-group differences, stereotyping, and discrimination, particularly in the labour and housing markets generating financial and social problems. Practical examples of these problems again reinforce stereotyping by the majority group, increasing discrimination further. As Hällsten et al. (2013) conclude, socio-economic resources and neighbourhood segregation can explain most differences. Following the contact perspective, social and crime-related problems increase the likelihood of negative contact in Sweden compared to the other two countries.

The Scandinavian countries have historically been characterised as liberal and with high scores on the *Migrant Integration Policy Index (MIPEX)* (Huddleston et al., 2015), which is a standard measure of states’ policies on migrants. Although the MIPEX scores for the Scandinavian countries differ (Sweden 86 points, Denmark 49 points, and Norway 69 points), recent empirical data indicates that all three countries are considered very tolerant. Table 1 shows data from the European Values Study (2020)^2^, indicating high levels of trust toward foreigners in the Scandinavian countries. Sweden scores higher than Denmark and Norway when measured on the trust of people of another nationality. Combining the two top categories from Table 1, Sweden aggregates to 93.62%, Denmark 89.02% and Norway 86.79%.

**Table 1:** Trust in people of another nationality (in percent).

	Sweden (*N* = 1159)	Norway (*N* = 1105)	Denmark (*N* = 3280)
Trust completely	29.7%	17.47%	23.93%
Trust somewhat	63.85%	69.32%	65.09%
Do not trust very much	5.35%	11.67%	8.93%
Do not trust at all	1.04 %	1.54 %	2.04 %

The survey was conducted in 2017 (Sweden and Denmark) and 2018 (Norway).

This data forms an impression that discrimination is less likely to occur. However, such results are likely to say more about ideals and self-image than if discrimination happens in practice. Finally, there can also be differences between sports practices and the broader attitudes expressed in the European Values Study.

## Literature review

Discrimination research is usually focused on the labour market (e.g., Bertrand and Duflo, 2017; Bertrand and Mullainathan, 2004). Trying out for a soccer team is expected to include some of the same patterns as found in the hiring process. However, soccer try-outs in non-professional clubs involve fewer risks for soccer managers than human resources or management staff hiring employees to a firm. Nor is there any economic gain associated with taking part in an amateur non-profit soccer league, so this is not expected to be a factor in this setting.

Sports participation is increasingly viewed by politicians and decision-makers as a tool to achieve social integration, especially for underprivileged and minority groups (Vandermeerschen et al., 2015). Organised social activities, like those in sports clubs, can transcend cultural barriers among people from different backgrounds (Dietl et al., 2021; Nesseler et al., 2019). As such, participants in organised sports are more likely to meet other ethnic groups in a relaxed cultural setting (Krouwel et al., 2006). However, over the years, some researchers have become sceptical about the potential of social integration through sport (Walseth and Fasting, 2004).

Scholars such as Hübinette and Tigervall (2009), Sawyer and Habel (2014), and Tigervall and Hübinette (2010) find that ethnicity can be a solid societal boundary that is challenging to overcome, so we could expect to see some ethnic discrimination in our empirical data. In a study by Engh et al. (2017), a preference is found for recruiting players from countries with assumed cultural proximity to Scandinavian countries, indicating that barriers exist. According to the study, one motivating argument was a belief in cultural similarities relating to soccer in terms of superior discipline and tactical capacities among European players. In his research on people’s decisions to join and leave voluntary organisations, including sports clubs, Wiertz (2016) finds that native residents are less likely to join organisations with more minority members, while the opposite is true for minority groups. Nesseler et al. (2019) use a field experiment to examine access to social activities in Switzerland. Gomez-Gonzalez et al. (2020) replicated this approach in 22 European countries. Their results are especially interesting for the current analysis as they include the three Scandinavian countries.^3^ Their findings show that discrimination is clearly at play in relation to men’s soccer in the three countries.^4^ The above-mentioned studies illustrate that the interaction preferences predicted by most discrimination theories are likely also present in the sports context.

We are interested in whether discrimination occurs for women with foreign-sounding names asking to participate in a soccer try-out and, further, the role gender might play in practising discrimination (gender of the recipient coach). Gomez-Gonzalez et al. (2021) showed that men with foreign-sounding names seeking to join an amateur soccer team are often discriminated against. Still, women with a foreign-sounding name belong not only to one but to two types of social minority groups (foreign and gender), and the effects of this could be expected to reinforce each other (Beal, 1970; King, 1988). These types of additive effects are, however, problematic. Race and gender are found to intersect (Rosette et al., 2018), meaning that women and men from the same ethnic minority may face different types of stereotypes and discriminatory behaviour.

In the psychological literature, social dominance theory (SDT) (Sidanius et al., 1994), predicts that men are more likely than women to experience discrimination because men are more often perceived as a threat (Dahl and Krog, 2018; Navarrete et al., 2010). From the perspective of social cognition theory, cognitive categorisation and stereotyping happens to achieve more efficient information processing (Fiske, 1998; Krieger, 1995; Reskin, 2000). However, when decision-makers depend on out-group members, they cannot afford to take this shortcut; they need to obtain accurate information. The challenge facing some women’s clubs to recruit enough players to make up a team could help reduce discrimination against women in this context—simply because the decision-maker cannot afford to discriminate.

Which ethnic minority one belongs to can also be of importance for the level of discrimination and negative stereotyping. Studies have identified the presence of ethnic hierarchies (Hagendoorn, 1995; Hagendoorn and Hraba, 1987; Snellman and Ekehammar, 2005; Thijssen et al., 2021), which involves a social consensus about how attractive different groups are to interact with. The hierarchical ranking is related to cultural distance from the majority group and socio-economic status. Those belonging to a group at the top of the hierarchy will benefit from this situation. The development of negative stereotypes about out-groups with a low ranking in the hierarchy can be related to the need to justify these advantages (Duckitt, 1992). Low-status groups may use the same stereotypes to differentiate themselves from other low-status groups, further aggravating the situation.

## Presentation of methodology and materials

### Methodology

We identified 1141 amateur soccer clubs in Denmark, Sweden, and Norway and the corresponding e-mail addresses of coaches in those clubs. This comprises the universe of all female amateur football clubs in the three countries. We then randomly selected one team from each club if a club had more than one team (Map 1 in the appendix shows the geographic location of the clubs). This is because contacting the same club twice—with the same e-mail text—could seem suspicious and be considered spam.

Following the guidelines of the ethical committee of Norwegian University and Science and Technology, we approached each club with the following e-mail text (translated into Danish in Denmark, Norwegian in Norway, and Swedish in Sweden):

Subject: Trial practice

Hello,

I would like to take part in a trial training session with your team. I have already played at a similar level. Could I come for a trial training session?

Many thanks

Name

In line with previous research (Gomez-Gonzalez et al., 2021), we constructed five typically foreign-sounding names for each country’s three largest ethnic groups.^5^ It was essential to choose the top three ethnic groups for each country rather than the same three groups for all countries due to differences across the countries’ foreign populations. For example, people from Turkey are the largest foreign group in Denmark, but they are only the 17^th^ largest in Norway and the 10^th^ in Sweden. We choose the most frequent names for each group. Table 2 shows the names and the individual responses for each country.

**Table 2:** Names and share of positive responses (in parentheses).

Names	Denmark	Norway	Sweden
Native-sounding	Lærke Pedersen (73%) Frida Hansen (75%) Astrid Jensen (82%) Liva Nielsen (50%)	Ingrid Andersen (67%) Marit Larsen (92%) Kari Olsen (85%) Inger Johansen (77%) Anne Hansen (83%)	Amanda Eriksson (87%) Moa Nilsson (72%) Elin Karlsson (79%) Julia Johansson (83%) Emma Andersson (64%)
Turkish-sounding (Denmark), Polish-sounding (Norway), Finish-sounding (Sweden)	Ayşe Çelik (70%) Hatice Şahin (45%) Zeynep Demir (36%) Fatma Kaya (73%) Emine Yılmaz (75%)	Zuzanna Kamiński (83%) Julia Wiśniewski (62%) Lena Wójcik (85%) Maja Kowalczyk (62%) Zofia Nowak (77%)	Sofia Mäkelä (76%) Maria Nieminen (74%) Aino Mäkinen (76%) Olivia Virtanen (76%) Aada Korhonen (63%)
Polish-sounding (Denmark), Lithuanian-sounding (Norway), Iraqian-sounding (Sweden)	Zuzanna Kamiński (55%) Julia Wiśniewski (82%) Lena Wójcik (80%) Maja Kowalczyk (80%) Zofia Nowak (50%)	Emilija Vasiliauskas (77%) Austėja Petrauskas (69%) Viltė Jankauskas (31%) Gabija Stankevičius (85%) Liepa Kazlauskas (71%)	Marwa Khalil (76%) Tara Al-Jamil (48%) Saya Abdel-Rahman (63%) Saleen Al-Bayati (58%) Zilan Al-Zahawi (65%)
Syrian-sounding (Denmark), Somalian-sounding (Norway), Polish-sounding (Sweden)	Fatima Malki (67%) Donya Hussain (82%) Yara Ismat (67%) Amira Nasri (50%) Safaa Khalil (55%)	Fatima Aden (85%) Sadia Haji (77%) Khadija Abdullahi (92%) Sumaya Bashir (77%) Halima Ali (72%)	Zuzanna Kamiński (70%) Julia Wiśniewski (62%) Lena Wójcik (56%) Maja Kowalczyk (67%) Zofia Nowak (72%)

We randomly combined one name with one club, ensuring that the different regions (e.g., densely populated districts like Stockholm or Oslo) had the same probability of receiving each name. Each club was only contacted once. We also double-checked that one coach was not approached at different clubs.

We created Gmail accounts for each name. The e-mails were expedited over three days and categorised into positive responses, positive responses with additional questions, and negative responses for the period October 1–3, 2020. A positive reply could be “Yes, you can come to practice” or “We practice Monday and Wednesday. Feel free to drop by.” A positive response with additional inquiries could be “Which position do you play?” or “How many years of experience do you have?”. Negative responses (e.g., “No, you cannot come”) are almost non-existent. In this experiment, most respondents prefer not to respond compared to giving a negative response.

A positive response with additional inquiries is different from a positive response. Nonetheless, a positive response with additional inquiries is a first initiation. It represents the first contact between the club and the player. The player can build upon this exchange and discuss with the coach if this team or another team at the same club is appropriate. This is not the case for a negative response (Dietl et al., 2021; Nesseler et al., 2019). It does not create any further communication. Thus, we followed previous research and combined negative and non-responses.

In all countries, sport club activities were more or less back to normal despite the Covid-19 pandemic. We responded to each positive response within 48 h, informing the coach that the applicant was no longer interested.^6^

### Data

The response distribution by country is presented in Table 3. Sweden (10.2 million) has a larger population than Norway (5.3 million) and Denmark (5.8 million) [at the time of the study], which also corresponds with the number of women’s amateur soccer clubs. We ended up with a larger number of observations for Sweden than for the other two countries. Like male amateur football, the number of inhabitants in a country is not necessarily related to the number of clubs. Different factors (e.g., division, club size, gender and experience of the coach) play a role. Thus, it is not surprising that Norway has more clubs than Denmark (the geographical distribution of clubs can be seen in Fig. S1).

**Table 3:** Answer distribution by country.

Country	Response for	All positive responses	Negative response	*N*^a^
Sweden	All names	460 (69.17%)	201 (30.23%)	665
	Native-sounding names	123 (76.88%)	37 (23.12%)	160
	Foreign-sounding names	337 (66.73%)	168 (33.27%)	505
Norway	All names	195 (75.29%)	60 (23.17%)	259
	Native-sounding names	51 (80.95%)	12 (19.05%)	63
	Foreign-sounding names	144 (73.47%)	52 (26.53%)	196
Denmark	All names	136 (62.67%)	76 (35.02%)	207
	Native-sounding names	32 (69.57%)	14 (30.43%)	46
	Foreign-sounding names	104 (64.60%)	57 (35.40%)	161

A few responses were unclear (4 in Sweden, 4 in Norway, and 5 in Denmark).

^a^We performed a power calculation assuming a similar response rate difference for women’s teams compared to men’s, c.f., Gomez-Gonzalez et al. (2021). Thus, we understand that only response rate differences up to and above 0.15 will be statistically significant for Norway and Denmark. Accordingly, we interpret all results for Norway and Denmark cautiously.

**Fig. 1:**
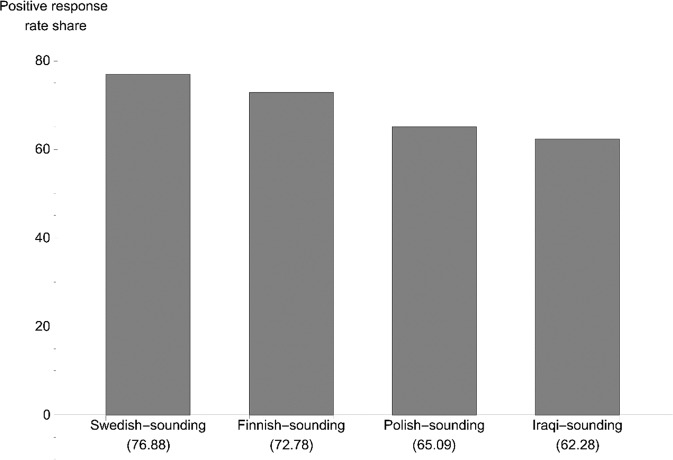
Percentage positive replies, Sweden (*N* = 665). *Note: N* for the respective names are Swedish (160), Finnish (169), Polish (169), Iraqi (167).

**Fig. 2:**
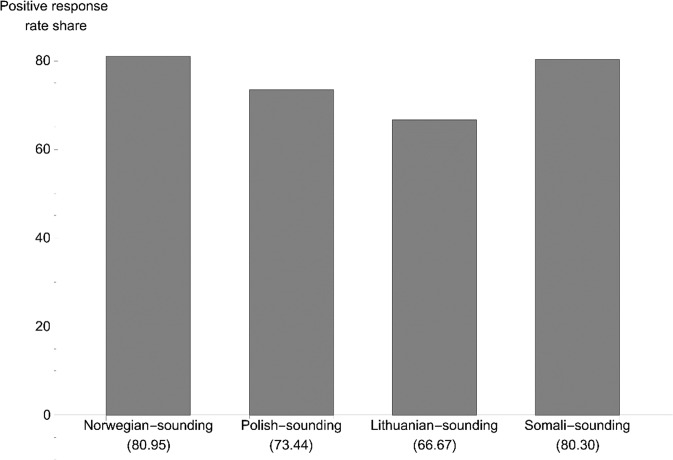
Percentage positive replies, Norway (*N* = 259). *Note: N* for the respective names are Norwegian (63), Polish (64), Lithuanian (66), Somali (66).

**Fig. 3:**
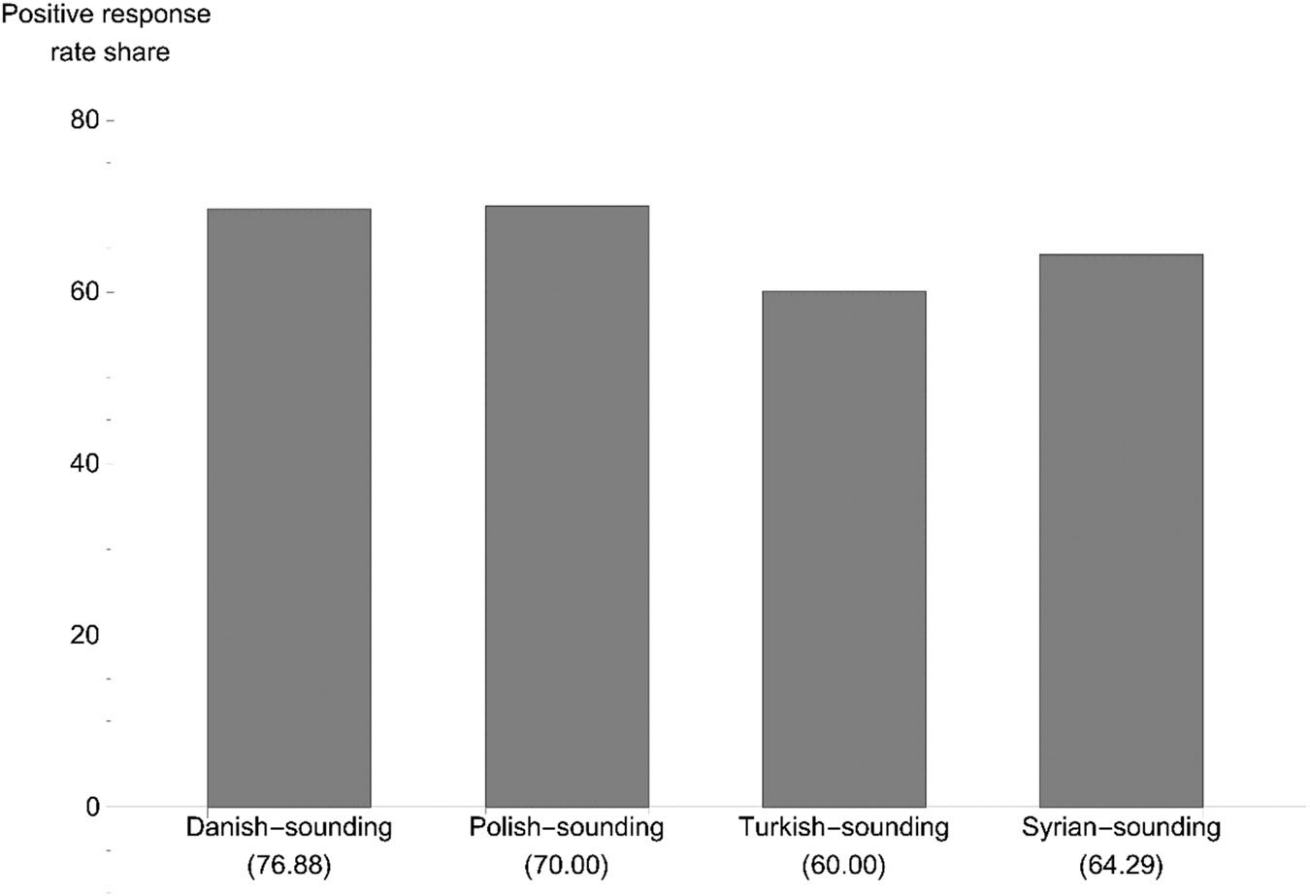
Percentage positive replies, Denmark (*N* = 207). *Note: N* for the respective names are Danish (46), Polish (50), Turkish (55), Syrian (56).

The positive response rate was the highest in Norway and the lowest in Denmark. We see that the response rate generally is higher for native-sounding names compared to foreign-sounding names in all three countries, with a 10.15 ppt difference in Sweden, 7.48 ppt in Norway and 4.97 ppt in Denmark. Comparing our response rates to those in male amateur soccer (Gomez-Gonzalez et al., 2021), the positive response rate for female football-players is generally higher for all countries and categories, and the gap in percentage between native-sounding and non-native-sounding names points is smaller for women than for men. In Figs. 1, 2, and 3, we present the percentage of positive replies for each country, arranged by the country of origin from which the request for a trial was sent. For Sweden, we see that the native Swedish-sounding names had the highest positive response rate (76.88%), followed by Finnish-sounding (72.78%), Polish-sounding (65.09%), and Iraqi-sounding (62.09%) names. In Norway, the native-sounding names scored the highest (80.95%), followed by Somali-sounding (80.30%), Polish-sounding (73.44%), and Lithuanian-sounding names. In Denmark, the Polish-sounding names scored highest (70.00%) together with native-sounding names (69.57%), followed by Syrian-sounding (64.29%) and Turkish-sounding names (60.00%). Native-sounding names received the fewest responses.^7^

### Regression approach and empirical models

We deploy a logistic regression approach to test whether foreign-sounding names are more likely to receive no or negative replies than native-sounding names (using OLS with a binary dependent variable has no significant influence on the results). In total, we present six regression models, two for each country.

The dependent variable in all models is a *positive reply* (0–1), where the reference categories include all non-answers and the limited number of negative and unclear answers. In Table 5, we test the effect of having a *foreign-sounding name* (with native-sounding name as the reference category); in Table 6, we distinguish the different sub-groups of foreign-sounding names within each country (see Table 4).

**Table 4:** List of name categories by country.

Sweden	Norway	Denmark
Swedish-sounding	Norwegian-sounding	Danish-sounding
Finnish-sounding	Polish-sounding	Polish-sounding
Polish-sounding	Lithuanian-sounding	Turkish-sounding
Iraqi-sounding	Somali-sounding	Syrian-sounding

We control for the gender of the person to whom the e-mail was sent through the variable *woman*. Female coaches are a minority in soccer (Gomez-Gonzalez et al., 2019), also in Scandinavia. The fact that female coaches are a minority is also evident in our samples, where 22.71% (Sweden), 19.69% (Norway), and 15.28% (Denmark) of the respondents, respectively, were women. We also include *division* as a control (the level of the team in the hierarchy of the series).

The logistic regression model provides the calculated probability of the dependent variable having the value 1 (positive reply), given the values on the explanatory variables, estimated using maximum likelihood. We examine the whole population of amateur women’s senior soccer clubs in the three countries rather than a sample. In this sense, we generalise from observations to the process or mechanism that brings about the collected data (Gold, 1969; Henkel, 1976). We see our experiment as non-deterministic, thus suggesting that the findings may vary even when holding the experimental conditions constant. Based on this, confidence intervals and significance levels make sense, even when investigating the whole population. From this perspective, the lack of significance suggests that the association produced by nature is no more probable than that produced by chance (Gold, 1969).

## Results

The regression output is presented in Tables 5 and 6. In Table 5, we see a significantly negative effect of having a foreign-sounding name on the likelihood of receiving a positive reply in the Swedish sample. According to our model, players with Swedish-sounding names have a 76.88% probability of achieving *Y* = 1, whereas a foreign-sounding name has a 66.73% probability. In Norway and Denmark, the effect of having a foreign-sounding name is negative and insignificant. Sending an e-mail to a female recipient is negative in all models but only significant at the 10%-level in the Norwegian model. We also tested the interaction-related term *woman* with *foreign name* but found no interaction effects. The control variable *division* was not significant in any models.^8^

**Table 5:** Logistic regression with foreign-sounding names.

	Dependent variable: all positive responses (positive = 1)		
	Sweden	Norway	Denmark
Applicant has a foreign-sounding name	−0.506** (0.210)	−0.473 (0.367)	−0.228 (0.364)
Coach is a woman	−0.042 (0.202)	−0.620* (0.341)	−0.482 (0.392)
Division control	0.030 (0.079)	−0.180 (0.214)	−0.050 (0.154)
Intercept	1.060** (0.446)	2.329** (0.944)	1.162 (0.833)
Observations	665	259	207
Log-likelihood	−407.645	−141.921	−132.098
Pseudo *R²*	0.008	0.020	0.008

**p < 0.05; *p < 0.10.

**Table 6:** Logistic regression with minority groups.

	Dependent variable: all positive responses (positive = 1)		
	Sweden	Norway	Denmark
Applicant has a native-sounding name	Omitted	Omitted	Omitted
Applicant has a foreign-sounding name and is from minority 1 group	−0.219 (0.255)	−0.470 (0.433)	−0.000 (0.447)
Applicant has a foreign-sounding name and is from minority 2 group	−0.577** (0.247)	−0.804* (0.424)	−0.419 (0.426)
Applicant has a foreign-sounding name and is from minority 3 group	−0.700*** (0.246)	−0.093 (0.454)	−0.233 (0.429)
Division	0.027 (0.079)	−0.190 (0.380)	−0.044 (0.155)
Coach is a woman	−0.027 (0.203)	−0.608* (0.344)	−0.466 (0.394)
Intercept	1.071** (0.447)	2.366** (0.952)	1.131 (0.838)
Observations	665	259	207
Log-likelihood	−405.395	−140.366	−131.584
Pseudo *R²*	0.013	0.031	0.011

***p < 0.01; **p < 0.05; *p < 0.10. For Sweden, Minority 1 = Finland; Minority 2 = Poland, Minority 3 = Iraq. For Norway, Minority 1 = Poland, Minority 2 = Lithuania, Minority 3 = Somalia. For Denmark, Minority 1 = Poland, Minority 2 = Turkey, Minority 3 = Syria.

In Table 6, we obtain a more nuanced picture of the effect for each group. For the Swedish model, the impact of having a Finnish-sounding name compared to the reference category (Swedish-sounding) is negative but not significant. A Polish-sounding name is negative and significant at the 5%-level, and having an Iraqi-sounding name is negative and significant at the 1%-level. The likelihood of receiving a positive reply for each of these groups is 76.87% (Swedish-sounding), 72.27% (Finnish-sounding), 65.12% (Polish-sounding), and 62.28% (Iraqi-sounding).

For the Norwegian model, the only significant effect (at the 10%-level) is the negative effect of having a Lithuanian-sounding name juxtaposed with having a Norwegian-sounding name. None of the effects in the Danish model is significant. We should remember that the Norwegian and Danish models have a considerably lower number of observations than the Swedish model.

## Discussion

The results from the Swedish sample reveal that foreign-sounding names from group 2 (Polish-sounding) and group 3 (Iraqi-sounding) received significantly fewer positive responses than the Swedish-sounding names. The negative effects on the responses also increase with geographical and cultural distance and are most evident for the Iraqi-sounding names, which corresponds with the theory of ethnic hierarchies (Hagendoorn, 1995; Snellman and Ekehammar, 2005). In contrast, our analyses show no statistically significant effects of discrimination against female minority soccer players by amateur clubs in Denmark and Norway. However, the small sample sizes in Norway and Denmark limit our interpretation of the results. On the other hand, male soccer players are subject to this kind of discrimination in the same countries (Gomez-Gonzalez et al., 2021), which suggests that there might be a difference in discriminatory behaviour towards men and women representing ethnic minorities attempting to join soccer clubs. It should be noted that the effect of foreign-sounding names is negative, considering the smaller samples from Norway and Denmark.

Compared to the study of men’s soccer, the total response rate for both natives and foreigners is higher among women’s teams. If coaches depend on an out-group member to complete their team, they cannot afford to discriminate and are more likely to make their decisions based on accurate information about the player’s technical skills. They should therefore be more likely to accept the player for trial practice (Reskin, 2000).

Sweden is the only country where foreign-sounding names receive significantly fewer positive responses. If discrimination is causing overrepresentation of minority groups in terms of social and criminal problems, observations of these problems, negative stereotypes, and discrimination mutually reinforce each other. This will be more evident the larger the relative size of the minority groups. Quillian et al. (2019) also find that Sweden is the second most discriminatory country (behind France) in their meta-study on discrimination in the labour market. Our findings from Sweden point in the same direction as other studies, which note that the Scandinavian norm of racial avoidance and self-perception as being “colour-blind” (Adjepong and Carrington, 2014; Engh et al., 2017; Goldberg, 2006; Hübinette and Tigervall, 2009) could be disguising the discriminatory practices that occur in everyday life.

Lastly, we do not find large differences in discriminatory behaviour among male and female soccer coaches. Among the coaches in Norway, women are slightly more discriminatory than men. The findings on gender differences in discriminatory behaviour suggest that these differences are affected by context.

## Conclusion and future research

In this paper, we tested whether women with names representing ethnic minority groups in Scandinavia experience discrimination in amateur soccer. Sport is chosen as our experiment’s laboratory because it is a third-sector arena that can foster social inclusion. Research indicates that discrimination also happens in sports, including soccer, even though most research is conducted in the labour market. So far, little research has addressed discrimination against women from ethnic minorities in sports.

Our results show that the probability of receiving a negative response to an enquiry about joining a soccer trial increases with geographical and cultural distance in Sweden. However, distance has less influence in Norway and Denmark. Seen in relation to existing research, our results indicate differences in discrimination patterns across gender. Earlier studies have indicated that discrimination exists in men’s soccer in the included nations. Overall, we suggest that there might be differences related to both country and gender, with the caution that sample sizes for Norway and Denmark are small.

Many illuminating perspectives can be derived from our findings. Most importantly, the results reveal the potential for minority women to achieve social integration in soccer clubs, at least in Denmark and Norway. This is a positive finding that could reduce cultural barriers across majority and minority groups because clubs allow people to meet in an informal environment to participate in fun activities.

In relation to Sweden, it would be beneficial to understand the reasons behind the discriminatory patterns to help counteract them and reinforce the potential for sport to encourage social inclusion. Being conscious of this tendency is extremely important in reducing the negative consequences of such processes. Creating more situations that foster positive contact between groups will counteract negative stereotypes. Employing more formalised procedures and composing heterogeneous decision-making groups regarding ethnicity and gender are relevant examples of organisational efforts that could help reduce discrimination (Reskin, 2000). However, eliminating discrimination and stereotyping is extremely difficult, and there is a need for more research on effective ways to address these problems (Wynn and Correll, 2018).

### Limitations and future research

The research conducted for this paper was limited by geography, sample size and a focus on selected ethnic groups, which paves the way for future research. First, we focus exclusively on the three largest foreign groups in the included nations, so it is reasonable to expect that other groups could experience more (or less) discrimination than those included. For example, foreign groups with a greater cultural difference from the native population than those represented in this study could be prone to more discrimination.

Second, the experiment focuses exclusively on soccer. Several other sports (e.g., handball, tennis, or athletics) are also popular and could be influential when it comes to social integration in these countries. It would be interesting if future research could compare results regarding discrimination in, for example, team sports and individual sports to test the generalisability of these findings. This would help to understand better discrimination practices than those demonstrated in this study.

## Supplementary information

Figure S1

## Data Availability

The data supporting the findings from this study are available at 10.7910/DVN/BCWQXI. Following the conditions of the ethical committee of the Norwegian University and Science and Technology, after the data collection ended, we deleted all the information that identifies individual responses.

## Footnotes

1 Data is available here for Norway: https://www.ssb.no/en/befolkning/artikler-og-publikasjoner/14-per-cent-of-population-are-immigrants, for Sweden: https://www.scb.se/en/finding-statistics/statistics-by-subject-area/population/population-composition/population-statistics/, for Denmark: https://www.dst.dk/en/Statistik/emner/borgere/befolkning

2 https://europeanvaluesstudy.eu/

3 The data from this study are publicly available.

4 It should be noted that there are some differences between how this experiment was conducted and how the study in this paper was conducted. First, the number of observations made is significantly larger, as there are many more male than female clubs in all three countries. Second, the minorities identified are not the same as in the current experiment (e.g., in Denmark, the second minority is indicated by German-sounding names rather than Lithuanian-sounding names). Gomez-Gonzalez (2021) use data from the CensusHub2 data, which provides information for all foreign groups and for all countries in their analysis. However, the data is outdated for some countries. Thus, in this analysis, national administrative data is used to define the largest foreign groups.

5 An exception was made for Denmark, where we did not include German-sounding names as they can easily be confused with Danish ones.

6 First, this was appropriate as the respondents might otherwise wait too long. Second, this was a condition of the experiment that was demanded by the ethical committee of the Norwegian University and Science and Technology.

7 We discovered problems with one of the Danish accounts (Freya Andersen) during data collection. The Gmail account was unable to receive responses and/or send e-mails. We could not detect the specific problem as we closed all accounts after the experiment. Therefore, the data from this account was not included in the analysis.

8 We also tested a dummy set of the different divisions, but this did not affect the results.
