# Supplementary figures and images for: Ethnic discrimination in Scandinavia: evidence from a field experiment in women’s amateur soccer
Source: Humanit Soc Sci Commun. 2023 May 11;10(1):230. doi: 10.1057/s41599-023-01734-7 (PMC10173913; doi:10.1057/s41599-023-01734-7)

**Figure S1.** Women’sAmateur soccer clubs in Denmark, Norway, and Sweden.


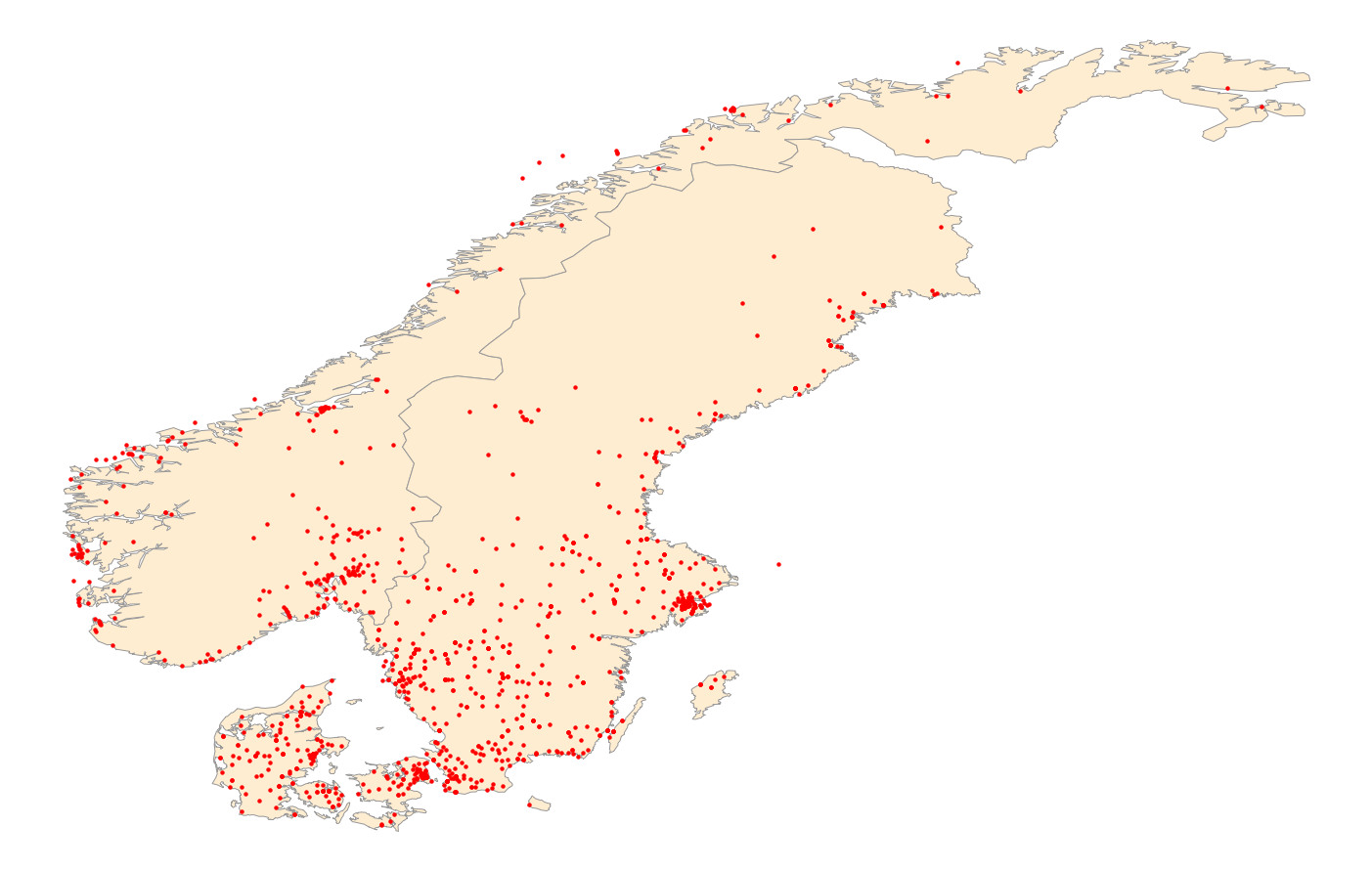

Supplement: Supplementary file 1 — Figure S1 [file 41599_2023_1734_MOESM1_ESM.docx]
